# The gravity of the status quo: the response of research governance to system-level shocks

**DOI:** 10.1007/s10734-024-01309-8

**Published:** 2024-09-26

**Authors:** G. E. Derrick, J. Robson, A. Oancea, X. Xu, M. R. Stan

**Affiliations:** 1 Centre for Higher Education Transformations (CHET), School of Education, University of Bristol, 35 Berkeley Square, Bristol, BS8 1JA UK; 2 Centre for Global Higher Education, Department of Education, University of Oxford, 15 Norham Gardens, Oxford, OX2 6PY UK; 3 Centre for Skills, Knowledge and Organisational Performance, Department of Education, University of Oxford, 15 Norham Gardens, Oxford, OX2 6PY UK; 4 School of Economics and Management, Università Carlo Cattaneo – LIUC, C.so Matteotti, 22 - 21053 Castellanza (VA), Italy

**Keywords:** Research governance, Research systems, Research culture, COVID-19, Pandemic response, Inequalities

## Abstract

Using interviews with global research stakeholders, this research explores how stakeholders within research-system-level research governance organisations conceptualised, responded to, and reasoned the realities of disruption caused by the COVID-19 pandemic, and how they positioned procedural changes to their governance mechanisms. Given that system shocks present critical challenges to established practices and embedded institutional norms, we use neo-institutional theory as a heuristic device to examine the relationship between the exogenous shock of COVID-19, trajectories of institutional norms and cultures, and the role institutional stakeholders play in managing responses. Across all the research systems studied (with particular focus on the UK, Australia, Norway, New Zealand, Hong Kong SAR, and Italy), participants were concerned about how the shock provided by COVID-19 had both revealed and entrenched deep inequalities inherent in their research systems and globally. There were tensions in how participants centralised the concept of the ‘normal’ as part of a process of recovery permeating all system-level responses, often with a sense of *wistful affection* for pre-pandemic structures, modes of operation, and embedded norms. Aspirations for short-, medium,- and long-term plans for research change echoed a dependency on returning to ‘normal’ and an inevitable pull of the norms of the pre-pandemic status quo. Despite the desire to ‘build back better’, the pull of institutional norms and the gravitational force of the status quo appeared too strong for meaningful change in recovering research systems.

## Introduction

Shocks have the power to destabilise existing systems, challenge organisational status quos, and change embedded institutional norms. A range of studies have already examined the impact of the COVID-19 disruptions on research, focusing particularly on research production, researchers’ experiences (Amano-Patiño et al., 2020; Cai et al., 2021; Lee & Haupt, 2021), collaborations and collaborative teams (Fry et al., 2020), novelty (Liu et al., 2020), and in universities (Porter & Hook, 2020). However little research has taken a systems-level approach by examining the relationship between COVID-19 disruption and research systems (which we conceptualise as dynamic configurations of institutions, organisations, and stakeholders involved in the governance, conduct, sharing, monitoring, and utilisation of research). Considering the position of research stakeholders, industry and governance organisations in the immediate to mid-term wake of the COVID-19 pandemic can assess how the sector, as an ecosystem of academic, government, and non-academic agents, will position itself to recover from the destabilising shock of COVID-19. There has been little research that takes a global perspective, acknowledging that COVID-19 disruptions may be operationalised differently in different country contexts and in different international organisations, and at different times during the 2020–2022 period of the pandemic emergency.

Crisis science and disaster science provide conceptual accounts of system-level processes of change, where normal scientific practices (in the sense of Kuhn, 1996) are altered due to an exogenous shock (Borup et al., 2006; Machlis & Ludwig, 2014; Nilakant et al., 2016; Shrum, 2010; Tierney, 2007) and normal regulatory systems are transcended in order to protect the science system while simultaneously mobilising it towards producing specific, crisis-focused societal benefits. Similar to post-normal science (Funtowicz & Ravetz, 1993, 2018) which is characterised by high-stakes decision making and system uncertainties, crisis science emerges after a period of ‘focusing events’ (Greenland & Fabiani, 2021) and a transition that is characterised by an approach to problem-solving inquiry, decisive analysis, and an increased interest by a broad set of stakeholders in research outcomes (Leonelli, 2020). For the purposes of this paper, research is depicted as dependent on an ecosystem of actors that involve practicing researchers, funding councils, policymakers, professional associations, business, government ministers, and organisational leaders (including but not restricted to Higher Education Institutions — HEIs). Post-normal science necessitates the creation of new epistemic structures that prioritise the involvement of all those that have a stake in the issue, through enhanced participation and epistemic inclusion (Funtowicz & Ravetz, 2018). Therefore, the involvement of these ‘research stakeholders’ in the response to the COVID-19 disruption experienced by the research system is both vital and currently unexplored.

The lens of neo-institutionalism can also be used to examine how, despite the aspirations of individual stakeholders to change research systems, the pre-existing norms and scripts embedded within research systems and system-level cultures, ultimately pull aspirations back to pre-pandemic norms. Any form of institutional or system-level change, while aspirational, struggled to be conceptualised meaningfully at an operational or practical level in the face of normative institutional and research cultural pressures. This paper uses neo-institutional theory (Alvesson & Spicer, 2019; Meyer & Rowan, 1977) as a heuristic device to frame and discuss these tensions, the relationships between individuals, and the pull of institutional norms. Specifically, it examines the intersection between the exogenous shock of COVID-19, the institutional norms and scripts embedded in research systems and their broader contexts, and the role of individual stakeholders in shaping system-level responses.

## Literature review

Crises present the scientific community with unusual demands, including the need for rapid solutions. Research, as an interaction between the practice of producing knowledge and the various governing stakeholders that fund and ensure the integrity of knowledge, is not immune to endogenous and exogenous shocks. Rarely has there been a global shock to systems, institutions, and their structures and processes equivalent to COVID-19. Whereas crises within national contexts, such as the Syrian war (Greenland & Fabiani, 2021), the Fukushima disaster (Greenland & Fabiani, 2021), or natural disasters (Campos et al., 2018; Rotolo & Frickel, 2019), may influence one context or a region of an otherwise global knowledge endeavour, COVID-19 was experienced globally, shaped by the different responses exercised by different nations. Responses, regardless of their nature, resulted in a range of significant implications for research, including decreased mobility and isolation of individuals, impacting global, national, and even local patterns of collaboration (Fry et al., 2020; Lee & Haupt, 2021); the increased incidence of homeworking along with increased responsibilities for caring (Lambrechts et al., 2021) home-schooling and maintaining familial wellbeing, clashing with the production and dissemination of science ‘normally’; the expansion and concentration of financial resources to projects positioned to immediately deliver responses to address the COVID-19 crises (Porter & Hook, 2020; Wagner et al., 2022), and the associated funnelling of funding to disciplines linked with providing solutions; the redirection and re-innovation of research methodologies that previously relied on human, or else face-to-face interaction (Liu et al., 2020; Zajdela et al., 2021); and the transition to online activities.

These studies highlighted how the pandemic disrupted the otherwise ‘normal’ way in which research was produced, shared, and evaluated, but so far, research has focused explicitly on the experiences of individuals or the dynamics of research within organisations (primarily universities). However, these experiences and organisational dynamics sit within broader institutional frameworks of national and regional research governance systems. Research as ‘normal’ is shaped by institutional frameworks, and COVID responses, while often operationalised at the organisational level and manifested in individual experiences, were established at a strategic system level within central research institutions and enshrined in central regulatory mechanisms.

This paper focuses explicitly on these system-level institutional structures and their relationship with research organisations and researchers. Taking this approach acknowledges that understanding the disruption experienced by individual researchers and universities is only part of understanding how COVID-19 disrupted and altered research practice. A system-level, institutional approach, provides key insight into understanding the post-pandemic trajectory of research systems and the institutional-level factors that shape organisational and individual behaviours.

System-level, institutional lenses provide insights into broader global research dynamics. Whereas individual research organisations and universities play a role in national civil societies, and therefore respond to crises on national levels, research is also a global activity that transcends national boundaries. System-level policies, approaches, and processes interact globally, mutually shaping each other through informal processes of isomorphism, overt policy borrowing, or economic and geopolitical tensions (Marginson, 2022b). For example, the restriction of research practice in one country or region context can have a follow-on effect to the practice of research in other countries that are experiencing COVID-19 restrictions differently. This is particularly poignant given the increased internationalisation of research teams (Chen et al., 2019), as well as considering that access and the use of knowledge are not confined within country-negotiated boundaries.

Therefore, the consideration of shocks on the research system extends beyond national-specific experiences of *pandemia* (Watermeyer et al., 2021) within universities, or even nations, to a place where knowledge still flows across borders but is limited in the way that people can interact to create this knowledge. For this reason, studies of the effects of COVID-19 on research have chosen to concentrate on measures of collaboration (Cai et al., 2021; Fry et al., 2020; Wagner et al., 2022), as well as the production of knowledge which is relatable to the individual scientist who is still reasonably able to communicate within teams, assuming that they are not personally affected by COVID-19 lockdowns. In turn, system-level strategies are influenced by responses from transnational and multinational organisations, and large-scale international research sponsors, publishers, and infrastructure providers. This illustrates the ways in which global dynamics shape system-level research institutions as well as the organisations and individuals that comprise them.

This research adopts the position that research, as simultaneously a human, organisational, and institutional exercise, goes beyond individual, organisational, and national boundaries and operates as part of a dynamic process of interactions across macro (system/institutions), meso (organisations, particularly universities), and micro (individual researchers) levels and cutting across local, national, and global contexts (as a ‘glonacal’ enterprise, see Marginson, 2022b; Marginson & Rhoades, 2002).

### Neo-institutional framing of research systems

Neo-institutionalism is increasingly applied in studies of HE (e.g. Robson, (2022) or applying DiMaggio and Powell’s, (1983) concepts of normative, coercive, and mimetic isomorphism in analysing universities, see Hüther and Krücken (2016), but has been used only rarely in the rapidly developing field of research on research (e.g. Frank & Meyer, 2020). However, key ideas from NIT provide useful lenses for examining the exogenous shock of COVID-19 and the implications for change to research systems.

At the heart of neo-institutionalism, as argued by early theorists (see Meyer & Rowan, 1977; Zucker, 1977; DiMaggio & Powell, 1983), is the view that the structures and practices of organisations are largely shaped, not by rationality or functional purposes (i.e. efficiency or effectiveness), but by ‘ceremonies’, ‘rituals’, and embedded norms that provide a ‘sheen of legitimacy’ (Alvesson & Spicer, 2019, p200). These institutional norms or scripts, embedded at the institutional or system level, are described as having ‘a taken for granted quality’ (Meyer & Rowan 1977) and can be defined as rules governing behaviour in social interactions that are sustained by shared expectations of compliance and sanctioning (Bicchieri, 2006).

Such a perspective emphasises that actors within organisations comply with institutional norms, following behavioural routines that reflect institutional habits (Meyer & Rowan 1977) and individual agency is viewed, at least partially, as a product of culture. Thus, while norms or scripts have the potential to orient individual behaviour towards socially or organisationally beneficial outcomes, they also can support and legitimise unpopular, inequitable, or even dysfunctional social or organisational norms and scripts. These can be seen as providing the mechanism by which institutions maintain stability over time. This underpins the concept of path-dependency, the idea that decisions and outcomes from the past mean that institutions follow trajectories that are inherently difficult to alter, resulting in consistent reinforcement and institutional inertia. Within this framework, institutional change is largely seen as being tied to exogenous shocks that produce critical junctures, leading to the creation of new paths.

In contrast, a number of sociological neo-institutionalists have aimed to provide a more nuanced analysis of institutional change, rejecting the view that exogenous shocks are the only mechanism by which change occurs. Mahoney and Thelen (2010), for example, argue that institutions actually evolve incrementally and endogenously, driven by agentic strategies of internal institutional actors leveraging existing ambiguities as part of ongoing processes of change. Within this NIT framework, change occurs despite the absence of exogenous critical junctions disrupting path dependencies, occurring cumulatively, but resulting in significant institutional transformation over time (e.g. see Thelen, 2004; Mahoney & Thelen, 2010; Hall, 2010).

System-level research governance can be viewed as a key institution, containing its own norms, scripts, and path-dependencies that shape organisations (including universities) and individual researchers comprising the system (Immergut, 1998). In research systems, centralised, system-level structures fundamentally shape the norms and scripts embedded within research organisations through setting evaluation standards, research funding allocation, facilitating the translation of research into societal outcomes, promoting international exchange and collaborations, and fostering researcher careers. All these activities and regulatory mechanisms can be seen as path dependent and containing embedded norms and scripts, reproduced and reinforced through processes of socialisation, enforcement, and incentivisation (Meyer & Rowan, 1977).

COVID-19 presented a disruptive force to institutional norms and a potential critical juncture, especially when responses demanded different ways of operating. This presented a rare opportunity for a ‘rescripting’ of established institutional norms and the development of new pathways. Using NIT in this context provides a useful set of conceptual tools for thinking through the potential process of institutional change and the relationship between exogenous shock and endogenous iterative change processes in a way that throws into sharp relief tensions between structure and the agency of key institutional actors. This process was visible in multiple social, political, and economic institutions manifested in the call to develop ‘a new normal’.

This paper examines this brief window of disruption by focusing explicitly on how different research systems responded to the shock of COVID-19 and how stakeholders conceptualised the processes of recovery and navigated the process of ‘creating a new normal’ within the research system. NIT provides an important heuristic device for framing the process by which responses were conceptualised its influence on embedded institutional norms, the development of new pathways, and the capacity for the agency of individual research stakeholders to be exert a process of institutional change.

## Methods

### Participants

This paper uses stakeholder interviews conducted as part of the first phase of a multi-scale study of six research systems, situated within national, regional, and international contexts. The interviews were conducted between February and December 2022. Participants were based in various areas with different COVID responses (Mathieu et al., 2020). Some areas were under lockdowns, and some were ‘going back to normal’ or largely ‘back to normal’. A total of 69 stakeholders were interviewed across six systems (England, Norway, Italy, Hong Kong SAR, Australia, and New Zealand) and in inter-/transnational organisations. The six systems were deliberately chosen to allow comparisons and dialogues across different types of formal research assessment exercises, as well as different social responses to COVID (Mathieu et al., 2020). During interviews, several participants shared knowledge and insights about multiple systems and sectors, where they held leadership roles. In those cases, they were attributed to multiple systems and/or sectors (Table 1).

**Table 1:** Country association of interview participants

Systems	Number of interview participants
Australia	8
UK (including England/Scotland/Wales)	22
Hong Kong	6
Italy	7
New Zealand	7
Norway	12
International or transnational, including Africa, Europe, and America (including Canada, the USA, and Latin American countries)	16
Total (with overlaps)	78
Total number of unique participants	69

Participants were identified via a combination of initial purposive sampling (including the key domestic and international organisations related to research systems under study) and participant recommendations. They include senior representatives of ministries and other government agencies, politicians, major research funders, research assessment agencies and assessment panels, publishing industries, library bodies, learned societies, unions, associations and networks (including regional), advisory bodies, and research on research institutes.

### Semi-structured interviews

Interviews were conducted online, each over an hour in duration, with follow-up interviews to explore additional topics. Interviews were transcribed in full, sense-checked, and pseudonymised. Interviews and parts of interviews in another language (Italian) were translated using a back translation technique (Brislin, 1970).^1^ Participants were assigned to a group in light of their current main role. Given their level of seniority and experience, many participants had moved between groups over time or held/ were holding concomitantly several roles. Interviews sought to elicit insights in relation to each. The interview schedule covered topics related to the research function in the participants’ research system(s) and explicit questions about the challenges and changes in the research eco-system, the participants’ specific organisation, and their own practice due to the pandemic. Questions prompted reflection on any policies and/or initiatives aimed at facilitating research recovery in response to the pandemic.

### Analysis

Transcripts were analysed thematically along three levels (eco/systems, organisations, and people, including comparative analysis across countries and groups of stakeholders). Inter-coder checks were conducted between primary and secondary coders to increase the validity of resulting codes and interpretations.

Quotes were selected to illustrate themes, and the source of these quotes is representative of the level of disruption experienced within that country context (Mathieu et al., 2020).

## Findings

### Disruption, response, and recovery

The unique temporal situation of participants at the time of the interviews allowed them to: report events during and following the pandemic-related restrictions in specific country contexts; describe their initial objectives; reflect on effectiveness in the short term; as well as engage in a position of horizon scanning relevant to their organisational role in governing a research future. The initial open coding of the interviews showed distinct categories in which participants reflected upon the impact of the pandemic on their role within research. These were (1) Disruption, where the influence of the pandemic on normal research practice was acknowledged and described relative to organisational objectives; (2) Response, where participants outlined how organisations reacted to the immediate and short-term effects caused by the COVID-19 pandemic disruptions; and (3) Recovery, where participants remarked on how their organisations were planning change in the future as a result of the pandemic experience, or else conduct hypothetical thinking about what should/could be done to achieve change. For the recovery theme, there was a tendency for participants to confound this with themes of response which are elucidated in the below sections. Although themes are presented in the following sections, they were not discussed as a linear set of stages and the apparent linearity of their presentation is an analytical artifact.

#### Disruption

##### Acknowledgements but no direct experience of research disruption

Stakeholders expressed awareness of the effects of COVID-19 on ‘normal’ research practice that had impacted individual researchers. Specifically, participants were aware of how the changes to working environments (from face-to-face to virtual working) had disadvantaged some groups, with particular focus on gender disparities exacerbated by the pandemic, compared to others. This included how participants imagined the long-term challenges faced by these groups; however, this was solely problematised, but not translated into meaningful actions or else into impetus to address these disparities in the future.

*…* I know people use online methods.. [but] you don’t have the visibility that helps you build those networks and I do think that will affect women, it will affect young people in the coming years so I do think that would be interesting to watch how that plays out. UK6-202203

Here there was an expression of otherness to problems experienced by the research workforce, but there is a lack of a short-term plan; ‘I think that it is not clear what will be the new course’ *IT7-202,206.* Indeed, the participant’s interest in ‘watch(ing) how that plays out’ suggests the inevitability of the outcome requiring no immediate preventative measure but something to be addressed in the future when more information is known. In addition, ‘otherness’ was also expressed when reflecting on the effect that the pandemic would have on wellbeing (‘…the impact of the pandemic on PhD students and ECRs in particular was that they were becoming very isolated and was having a real impact on mental health so obviously that’s a negative aspect’ UK11-202,204); or else to disruptions caused by restrictions to certain lab-based scientists; ‘…[COVID-19 was] so not kind of wet labs which had to keep going…’ UK16-202,204.

For other participants, there was a caution against over-generalising the impact of disruption on research practice because of ‘…highly variable [disruption] across the community and across our sector, depending on people’s personal circumstances, depending on even which state they lived in.’ AU4-202,204. The reference here to the disruption depending on what state, refers to the pattern of restrictions in Australia, where each state implemented different COVID-19 measures at different times. This type of differential considers the effects of disruption on research, where the extent and timing of disruption differ. This reinforces the context- and system-specific comparison.

However, there was an acknowledgment that the disruption of COVID-19 pandemic exacerbated, not caused, inequalities of marginalised groups of researchers; ‘…COVID did the worst thing for the EDI [Equality, Diversity and Inclusion] agenda in the last two years that we could ever had imagined.’ *UK10-202204.* These interruptions and the potential long-term implications were recognised in relation to caring responsibilities (Derrick et al., 2022) and the capability of individuals perform in line with pre-pandemic expectations, with effects felt long term.

Thus, repercussions of the COVID-19 situation will influence a generation of policies aimed to target discrepancies highlighted by the pandemic, as a meaningful point of focus for a new normal in research. For participants, these exacerbations acted to highlight inequalities and to open potential avenues for action towards a more equitable research culture.

#### Response

Global disruption of the pandemic initiated calls to address a wide range of institutionalised inequalities, manifested in embedding cultural processes and methods of orientating and performing research work. Participants, working at the macro-level of national and transnational research systems, reflected on the potential of the pandemic to catalyse change:

I think the lessons that COVID taught us about flexibility, the unimportance of distance, the use of digital technologies, and the power of those things merge. UK21-202207.^2^ …the pandemic really pulled everyone together, and people put in extra effort, extra energy into it, and tried to get something done to help out in the situation. HK2-202202

Although this initial impetus for change was common across all participants, the mechanisms were conceptualised in a variety of ways by participants, revealing how research stakeholders positioned themselves towards initiating research culture change.

How organisations learn from experience is important in how they develop resilience against the possibility of future, exogenous shocks (Nilakant et al., 2016). Research, as an industry, enterprise, and culture are no different in this regard. The ability for organisations to develop ‘adaptive resilience’ characterises how they initially respond to a disaster, recover, and then renew themselves within a post-disaster environment. The role of leadership towards developing this resilience is to foster the well-being of employees, enable collaboration, and learn from the experience (Nilakant et al., 2016). For this argument, resilience is referred to here as a process rather than as an organisational trait. As research is an organised practice involving specialised practitioners and their ‘lifeline organisations’ which provide essential infrastructure services (Nilakant et al., 2016), how these organisations responded in the immediate aftermath and during the prolonged period of COVID-19 disruptions develops their resilience.

Within the responses, comparisons emerged that represent different needs of countries or regions from science during the crisis, depending on the type and timing of COVID-19 restrictions and disruption. In the above section, two common options were identified as available to governing stakeholders in response to the restrictions imposed by the pandemic: (1) make research work easier, or (2) promote and push research towards translation. The latter is a key characteristic of crisis science (Nilakant et al., 2016).

For most funder-stakeholders, the immediate response to the societal need of the pandemic was to increase the level, and concentrate the direction, of research funding towards ‘COVID-related issues’. This was even evident in countries, such as Norway, which, unlike e.g. the UK, were not most prominently participating in the vaccine race (Skjesol & Tritter, 2022). Other similar countries included Italy and New Zealand. For these countries, there was still a need for research that directly addressed locally based COVID challenges as an immediate policy response. In New Zealand, where COVID had comparatively little impact on individual or domestic restrictions (Mathieu et al., 2020), this urgency was reduced and research response de-prioritised in favour of non-pandemic domestic policies.

…a small place so we’ve only got five million people and we do a very small percentage of the research globally so it’s very important that the Chief Science Advisors and policymakers are connected to the international evidence base. NZ6-202208

Although there may have been nationally-based pockets of non-disruption because of COVID such as New Zealand, the pandemic affected the structure of global science (Marginson, 2022a, 2022c) to the extent that even these pockets felt changes to ‘normal processes’ of research. However, for New Zealand, the pandemic highlighted the importance of research locally; ‘…the pandemic has certainly, it’s had a number of quite positive effects…one in causing a re-evaluation of the importance of research.’ NZ5-202,207.

This action indirectly contributed to the centrality of research as an institution of societal trust, even in countries such as NZ where there was comparatively less disruption; ‘It is now much better understood that people who have work in universities do research, and then research can really help inform a whole range of activities in really important ways’ *NZ1-202,202.* At the same time, it acted towards furthering existing disciplinary disparities within research, highlighted as the ‘first consequence’ of the immediate response to COVID:

One of the first things that happened was that the Research Council made several calls for research-on-COVID and COVID-response... So, it has actually, the COVID has actually diverted a lot of funding to COVID-related issues, that - that is the first consequence NO2-202202

From the larger sample, an additional immediate response was to protect the institution and to sustain research throughout the pandemic. In addition, these concentrated on protecting the reliability of the knowledge produced, and on ‘keep[ing] things running as normally as possible, rather than to divert our funding into extending existing grants or doing something very different because of COVID’ AU4-202,204, or else mobilising (reliable) knowledge towards translation through, for example, increased need for open access knowledge:

…what that did for publishing is sort of helped to pull along a debate around open access is absolutely key and that’s still bubbling….so COVID did focus the mind around the need for open access publishing UK6-202203.

Here there was a conflict in how the institution, on the one hand, wanted to protect the reliability and robust nature of research, but, on the other, was faced with a research workforce that was temporarily unable to conform to the bureaucratic measures normally needed to govern this reliability. As one participant stated, in the ‘relax[ing] of reporting requirements and all sorts of other kinds of administrative requirements because we know people were finding those things very difficult to deal with when everybody was working from home.’ AU4-202,204.

Finally, despite an acknowledgment of increasing EDI disparities in the research workforce indirectly affecting the production of knowledge during the pandemic, less attention was paid to implementing changes, or else protectionist mechanisms to address these research practice concerns.

#### Recovery

Interviewer: What’s the trajectory of travel after COVID?

Participant: I really don’t know. (UK15-202,204).

The timing of the interviews, during the various COVID-19 lockdowns as well as during the immediate aftermath of the lockdowns in many countries (all post-vaccine) presented an opportunity for participants to conduct some hypothetical horizon scanning of the long-term effects of the pandemic disruption. Within these discourses, there was an assumption that the actions and support deployed by many research stakeholders as an immediate response to the pandemic were not sustainable in the long term. The return to ‘normal’ was posited as a necessary achievement prior to re-creating a ‘new normal’; *‘*I wish the COVID would be ended as soon as possible so everything could (sic) go back to normal’ HK3-202,207. Under this assumption, there is the danger that the return to normal would stymie achieving meaningful long-term research recovery*.* Therefore, there was an overarching dialogue that betrayed an assumption that the ‘new normal’ was only achievable via an initial return to ‘normal’ which made recovery ‘harder to talk about…’ *AU4-202,204.*

##### ‘Normal’ does not exist but we will get there

Participants frequently referred to ‘normal’, or a ‘new normal’ in their reflections of a time during the pandemic, or else envisioning a research future post-pandemic. Whereas this terminology is understandable considering the time of the interviews being in the immediate aftermath of COVID-19 disruptions, its use in relation to framing future plans beyond the pandemic revealed shifting reflections and re-constructions of understandings of ‘normal’ that shaped how organisations will formulate and evaluate their planned pathways to recovery. Without systematic recognition of the disruption and inadequate support to foster recovery, there is a danger of ‘normal creep’ by research governance that silences the legacy of research disruption and its effect on the workforce:

…we essentially just went back to how things were. So, lockdown measures were taken away. The restrictive measures were taken away [and] we were essentially expected to go back to everything as normal INT-AF2-202207.

Iterations of a return to ‘normal’ or else the visioning of a ‘new normal’ post-pandemic were made irrespective of acknowledging the drawbacks of pre-pandemic research systems; instead, various participants provided a vision of a new normal that was a cleansed version of the past.

The most immediate response among the participants was to protect and sustain the status of ‘normal’ science by orientating strategies towards regaining a normal where sustainable change could subsequently emerge. For participants, the desire for research change was confounded with an aspiration for the ‘normal’, sometimes framed as a ‘new’ normal. Nonetheless, change was regarded as necessary due to the level of disruption sustained when the interviews took place. One participant in Australia remarked, disruption needed to be transformed to meaningful change; ‘something good had to come out of it, because mostly it’s been bad for many, many people.’ AU1-202,203.

The path to research recovery, therefore, was concentrated on adapting to a mythical new normal that included the inclusion of a declarative normal (‘Actually, financially we did better during COVID years than normal’ UK14-202,204) as an aspirational pre-condition for recovery. When referring to ‘normal’ therefore amidst their discourse, their reference was associated with the past, and a new normal as moving towards the future.

This temporal association of ‘normal’ presented an effective construct for participants to use to outline plans for recovery. It also demonstrated a mechanism of governmentality in the way that participants used it to both place temporal constraints around recovery (‘We also made the decision to try to keep things running as normally as possible,’ AU4-202,204; or ‘adapting to the new normal…’ HK1-202,202) and also as a marker against which to evaluate the success, or at this stage potential success, of any envisioned change and/or change mechanism implemented to drive research recovery. There was a danger here that a short-termism governed by a naïve view of normal would mean that the lessons learned from COVID would ‘just be a bad memory’:

Higher education generally snaps back to the shape it was in before, so my suspicion is that research will do pretty much the same. Hopefully COVID will just be a bad memory. I hope so. UK15-202204

The discourse of a return to normal was also framed as a risk to driving meaningful, long-term change: ‘…I guess that’s the risk, is do we just go back to pre-pandemic practices, or have we learnt?’ *UK4-202,203*. Even though research recovery is a long-term process, the language of ‘normal’ was used to facilitate a vision for research recovery focused on short-term change mechanisms implemented in the immediate, or else visible future. An aspirational shift, rather than a process of long-term practical changes in how research operates that as one participant stated: ‘…because it’s just reality, what can you do about it? Do I think that it [COVID] will fundamentally change research? No, I don’t think so’ UK15-202,204*.*

Indeed, participants were adept at making comparisons between pre- (normal) and post- (new normal) pandemic working practices in research and drawing positives from the experience; ‘we were actually doing more than we would under normal conditions’ NO2-202,202. At the same time, such comparisons within discourse diminished the shock of COVID and indirectly dampened momentum towards systemic and sustainable change to research systems as a part of research recovery.

And while we all went online, so it was an organisational change of course, people worked very hard, so this is kind of a, we have this, it’s, you couldn’t call it Long Covid, but it’s a long-term, you know, fatigue that we have because we have been working under quite stressful conditions, and under those difficult conditions we were actually doing more than we would under normal conditions, so that’s how it has affected the Research Council, and I suppose also many other. I don’t know what will be the longer-term consequences on the research system. NO2-202202

The participant also refers to ‘fatigue’ as ‘Long-COVID’ suggesting that the depth of the crisis, and the demand of working under crisis-conditions, may act to further diminish any systemic appetite for further research change. A return to an imperfect normal, therefore, may be seen as preferable over the demanding task of major reforms.

##### Immediate change

The most immediate action towards recovery already planned was to implement policies that would future-protect the research workforce. This period is characterised by evaluating the effects of the pandemic against the idea of a normal research culture with a particular focus on what respondents discussed as the most immediate concerns. This stage was characterised by consultation and achieving winnable, public-facing solutions to address the disruption experienced. As such, the evaluation of the damage caused by the COVID-19 disruption is key to driving change at this stage.

Participants reflected on the immediacy of the stage and how it overlapped with the response stage, leaving very little ‘space’ for considering recovery as a separate stage; *‘*No space for recovery, still handling a crisis’ (AU2-202,203)*.* They reported a lack of forward thinking on behalf of research stakeholders about recovering research, due to the continual managing the crisis took priority: ‘We haven’t got time to think …. we just haven’t got time to think about it. We have to, you know, we’re managing a pandemic….’ AU2-202,203.

The approaches discussed highlighted the absence of both ‘knowing’ and of available tools to deal with the uncertainty brought on by COVID-19. Participants referred to actions that called for support (such as, in the case below, *‘*funds’), to understand the nature of the disruption before implementing interventions to ‘restart’.

I think another thing that we don’t really know how to deal with is the interruption of research, where research grants have simply run out and clinical trials or public health interventions or major experimental programmes have just had to stop. And the funds aren’t there for them to restart. AU4-202204

Approaches employed here focused on achieving measurable goals in the short term that can rectify the ‘damage’ of COVID rather than underlying difficulties of the system. For example, an Australian participant describes the challenge they face, as a funder, of how to acknowledge and accommodate ‘differential impacts of the pandemic on people’s productivity’ in evaluations:

*…* how do we make sure everybody’s productivity and track record is assessed fairly when there’s these huge differential impacts of the pandemic on people’s productivity? So, I think that’s a problem for us, as a funder. It’s a definitely problem for the sector. AU4-202204

While they acknowledge that this is a ‘problem for the sector*’,* it still centres COVID as a reasoning for implementing immediate response tools, rather than acknowledging that problems were amplified, and not created by COVID requiring long-term structural and governance changes. The participant acknowledges a lack of understanding or tools available, as well as of evaluations of the efficacy of such tools, to navigate this challenge in the short term and to make the outcome ‘work effectively and be fair’; ‘it will be very difficult to do. So, it’s very hard to know how to make that work effectively and be fair.’ *AU4-202,204.* There was a reluctance to initiate wide-ranging change mechanisms for global research systems, acknowledging that countries, institutions, and individuals would have experienced the disruption in different ways, and therefore would need different tools to enable recovery as a long-term process and strategy*.*

The above excerpts were predominantly taken from participants from Australia, which had a unique pandemic experience due to the devolution of the states and their differential COVID responses, and the country’s geographical isolation. Despite this, there was a sense that change in the immediate term was to be governed within individual stakeholder organisations (such as tenure extensions and bridging funding) but not by driving similar change instruments across the global research environment.

##### Medium term change

Discourse about change in the medium term focused on achieving winnable, outward-facing solutions as well as motivations to more broadly adopting established change movements already established within research culture (e.g. Open access; Responsible Research and Innovation—RRI; Impact and innovation, etc.). At this stage, change is not measurable as it is not the primary focus, as one participant described, and there is no certainty about what change is possible in the long term; ‘What I don’t know is actually if there are going to be long term changes’ *UK6-202,203.* Instead, there is a move to promote existing movements rather than creating new movements. Similarly, to the immediate level, there is little to no emphasis on the necessity to break existing structures, as opposed to using the normal.

As an example, open access, which had been a key topic of discussion prior to the pandemic, was exposed as a mechanism ripe for change post-pandemic: ‘so COVID did focus the mind around the need for open in publishing*’ UK6-202,203*. During the pandemic, there was unprecedented growth of the publication or pre-peer review preprints (Ioannidis et al., 2021) and the sharing of data and expertise beyond the confines of peer-review journals (Torres-Salinas, 2020). The participants reflected on how in the shadow of the pandemic the barriers towards its adoption had now been trivialised, moving the argument towards adoption rather than its merits and implementation barriers. As one participant reflected that the experience of the pandemic had pushed the overall benefits of the wider adoption of open-access and open-science principles:

I think we’re at the point now where, you know, everybody recognizes that open science and open access is a good thing, that we need it to happen. I mean, the pandemic has just, you know, finally laid that to rest. AU2-202203

The barriers to wider adoption, considered prior to the pandemic as insurmountable by the publishing industry (Fecher & Friesike, 2014) had been overcome by the necessity of the public health criterion (societal concern) and political will; ‘… publishers used to say that’s really hard to make stuff open, when they were really told that they had to do it by The White House, they kind of somehow managed to do it……so (laughing) it’s not that difficult, it’s just an issue of will’ *AU2-202,203.* The wider adoption and acceptance of the benefits associated with open access had also opened the door to the acceptance of other, parallel movements such as open science, data, and software; ‘open data and open software are the next things to develop, but I think open access is transformational for academia’* UK14-202,204.*

The instance of hybrid conferences was an initiative that was broadly adopted globally within the academic/university industry (Etzion et al., 2022). Though debatably a product of necessity brought about by the pandemic, the growth of virtual conferences was also fuelled by a wider concern regarding the sustainability of academic travel (Etzion et al., 2022; Lessing et al., 2020). Whereas, prior to the pandemic, there was an awareness of the issues of international travel and climate change, this had not fuelled an adoption of video conferencing so widely as to offer to rival in-person attendance.

… this change of being able to access people in different countries, you know, we’ve had video conferencing for absolutely years but we didn’t use it in the same way so the sort of conference scene I think has changed and may change forever, you know, the hybrid conference bit, the more selective piece which ties in with the sustainability piece. UK17-202204

The question remained as to how the widespread adoption of technologies to substitute in-person attendance would prove sustainable over time. Participants envisioning change in the medium term were driven by a move to identify (and in some instances quantify) the loss experienced to the institutional- or organizational-specific mission, and make moves to rectify these losses: ‘We’re still waiting to see because we don’t know the long-term consequences’ *NO5-202,203*. Recuperation was not focused on changing behaviour but on providing existing avenues that were focused on outcomes. There was a movement towards embracing ideas for wider, more systemic research culture change: ‘…the research council has a call that is going to look at the consequences of the pandemic on research so I guess that is the most concrete thing at the moment’ *NO8-202,203. I*n many situations, there was a caution towards initiating change mechanisms when compared to an approach that favoured taking the lead from other movements (such as Open Science or sustainable research practice). In all situations, evaluation and reflection were depicted as the essential first steps in this process of change.

##### Wishes for the future and long-term change

Fewer participants were comfortable engaging with levels of long-term thinking required to consider a research future beyond the immediate response to the pandemic and plans for research recovery in the short(er) term. This could have been due to the timing of the interviews (during 2022) where more reflection would have oriented recall of behaviours and strategies beyond those of managing the immediate aftermath of the pandemic. When discussing future plans, participants were reluctant to commit changes to research culture that necessitated a wider, systematic change to processes, procedures, and governance. There was a reluctance to initiate substantial action in the absence of sector-wide agreement on systematic change. Here, participants showed a dependency on the active engagement of other research stakeholders and an awareness of existing intra-institutional barriers e.g. ‘the absence of extra money’. There was a reluctance to engage in planning in the absence of cooperation from ‘other funders’ when the state of the system was still uncertain:

We are interested in how other funders are addressing those issues, in the absence of extra money. If funders have extra money, that’s great. But if you’re dealing with a fixed budget, it’s not so easy to know what you can do that’s special to help people bridge this gap. AU4-202204

At this stage of recovery, participants viewed the research process as messy and uncontrollable. It was implicitly assumed that stakeholders were beyond research practice, remaining separate without understanding their role of placing opportunities within the system. Although COVID had emphasised the frailty of research systems, there was a tendency to focus on changing research to achieve public-facing outcomes, rather than change the nature of research for research’s sake. In the below excerpt from Italy, change in research processes or governance would be focused on creating an environment where research benefits for society would be realised. The pandemic disruption in Italy had resulted in a reliance on ‘foreign vaccines’ arising from research conducted elsewhere, rather than producing a research environment within Italy that was capable of delivering its own societal outcomes. The pandemic highlighted an ‘incapacity to complete the change of research…and a lack of a strong research policy’, and jeopardising an argument in favour of research’s public value. This would be a focus on long-term research recovery:

But it has also demonstrated the weakness of the Italian research, or better, its incapacity to complete the chain of research and the distance between universities and companies and the lack of a strong research policy. The demonstration is in the fact that Italy has tried, but it has not managed to issue a vaccine on its own….in the end we received foreign vaccines. It’s not a nationalistic issue; it’s just a demonstration that the potential is high but with the situation, the results are not granted. IT2-202203

This tension between research practice and public value was also seen in Norway with doubt that sustainable change was possible, but opportunity for the pandemic to re-capture public trust in research processes and research institutions:

We need to communicate results more, I think. So, we need to use this opportunity. But for everything else, this is up and down, up and down. I think at the moment there’s a lot of trust in it because people have, yeah, you know, later on something else will happened and that will overshadow the pandemic. So, I think, yeah, at the moment we need to use the opportunity. But if it will sustain, I’m not sure…. NO1-202202

Despite this enthusiasm, there was limited understanding how this longer-term public trust in research institutions would be achieved. This outward-facing exercise was identified as the (feasible) goal, as opposed to instilling change in research practice and governance. There was a sense of long-term ongoing adaption, as opposed to more specific change of governance that could survive during the pandemic and continued in the long term; ‘I can see a lot more assessments actually taking place using virtual systems because, you know, it’s been demonstrated it can be done now.’ UK1-HK-202202.

One research governance institution that was highlighted in need of long-term systematic change was peer review. Participants challenged the role of peer review because of the pandemic which provided necessary environmental triggers to start questioning the power this process held in research. For example, the experience of the pandemic had demonstrated how ‘… the world didn’t fall apart when you put stuff out without peer review’ *AU2-202,203* and how the pandemic had led to questioning its necessity:

…the role of peer review I think is something that has been challenged during the pandemic…and we still don’t fully understand what the importance of peer review is and when you need it and when you don’t need it. AU2-202203

Breaking these governance mechanisms, however, was envisioned as a controlled event with buy-in from the wider research community and stakeholders.

## Discussion

This paper problematises how research stakeholders view their role in research recovery and re-constructing a ‘new normal’ following the COVID-19 pandemic. As initial actors of change within a broader, global research system, stakeholders play central roles in reorientating research funding and policies through altered performance targets for research funding, visibility, promotion, and evaluation. Their over-reliance on the concept of ‘normal’ as a threshold for recovery is questioned in relation to the types of systematic change required post-COVID-19 which will have long-ranging effects requiring consideration of how research policy may acknowledge and implement change to rectify damage to research as an institution. Findings show the frailty of ‘normal’ and ‘new normal’ as meaningful objectives to guide action, and the pandemic as a catalyst for rectifying long-term inequities institutionalised in research culture (Sugimoto & Larivière, 2023).

Participants recognised how the ‘shock’ of the COVID-19 pandemic had highlighted institutionalised biases and difficulties inherent in research systems globally, and not differentially related to the levels of lockdown severity. Early in the pandemic, when research stakeholders reflected on the research disruption due to pandemic-related restrictions, there was hope that an exogenous shock of this magnitude would act as a critical juncture, resulting in new pathways and a reformed research system. These echoed wider explicit aspirations expressed by a range of stakeholders to rebuild a stronger, kinder, and more equitable research system (Derrick, 2020). However, in neo-institutional terms, the dominance of pre-existing institutionalised norms, the strength of the pathway dependencies, the powerful forces of institutional inertia, and the discursive entanglement of institutionalised myths and scripts meant that the institutions remain both unchanged and unchallenged by the shock provided by COVID-19. Ultimately, despite, aspirations and discourses of change, any kind of grand vision for ‘the new normal’ gravitated towards a reproduction of the old.

Within institutionalised research systems, the power of path dependencies and embedded scripts and norms pulled organisational behaviours back to the status quo, even in the face of a significant exogenous shock (Meyer & Rowan, 1977). COVID-19 made visible the previously largely unquestioned pathways, structural limitations, practices, and inequalities in research systems (Amano-Patiño et al., 2020; Cai et al., 2021; Lee & Haupt, 2021). However, the force of patterned behaviour associated with research institutional norms can be seen as returning systems to previous unequal structures and practices despite discourses of change. The inequalities made visible by the pandemic have increasingly been rendered invisible once again as the power of the institutional script and historic pathways has once again become dominant.

Although the human aspect of crisis science was acknowledged when discussing how organisations responded to the pandemic, they did not attempt to alter their own practices, responsibilities, or positionality in the governance of recovery. Although more incremental understandings of institutional change emphasise the importance of the agency of institutional actors (Mahoney & Thelen, 2010), the unique place of research stakeholders to stimulate and drive sustainable change were underestimated in favour of short-term strategies focused on fragile constructions of ‘normal’. Implementation of possible change was dependent on how other research stakeholders in all countries would react in the medium and long terms, as well as retrospective evaluations of the extent of disruption. There were tensions between viewing researchers as passive recipients of change subject to a culture that determines the norms of research behaviour, and a wider ecosystem including research stakeholders who do not envision their role as having direct responsibility or power to initiate change.

No participants envisioned a role for researchers as change-actors within the research system. Instead, researchers are recipients of change and beneficiaries of a ‘new normal’. For participants, despite working at the institutional levels of the research system and being actively engaged in policy-making and regulation the responsibility for changing institutional norms or developing new pathways was viewed, inherently, as belonging to other parts of the research system. As such, a ‘new normal’ was almost always framed in passive terms as something that should be done, not in active terms as something to be owned and driven. This highlights the magnetic force of the institutional status quo, the strength of path dependencies, and the power that dominant norms within research systems can exert over the agency of individual actors within them — even in the face of a significant shock.

This illustrates the inability reported by research institutional stakeholders to sustain meaningful, long-lasting change in the research system. Their role remains as guardians and monitors of research, rather than as governors of responsible scientific practice. For this group, their responsibility did not extend to the micro level interactions in knowledge production but focused on maintaining the strategic and financial future of the institution, mobilising research translation focused on crisis-related scientific benefits, and ensuring future public and political support of publicly funded research practice.

COVID-19 was a profound shock to research as an institution. However, despite some of the most powerful stakeholders and institutional actors acknowledging the disruption as a critical juncture in the institution and an opportunity to develop new pathways and institutional norms, little institutional change appears to have taken place. The key actors felt disempowered and remained passive players in a process of change. This suggests that the institutional norms and path dependencies in research are so strong that few exogenous shocks could actually lead to meaningful institutional change. COVID-19 was not one of them. The inexorable power of the historic status-quo was too profound. However, although change is certainly desirable, this suggests that it must be incremental and iterative, driven by the individual agency of a far greater range of stakeholders within the institution at both system and organisational levels.

## Data Availability

Data is available on request. Applications for data will be considered on a case-by-case basis by the research team.

## Footnotes

1 Translated quotes used in the manuscript are noted with a *

2 Participants’ pseudonyms are structured as ‘System/Number-Interview year and month’.
